# Urbanization may reduce the risk of frost damage to spring flowers: A case study of two shrub species in South Korea

**DOI:** 10.1371/journal.pone.0191428

**Published:** 2018-02-07

**Authors:** Hyeon-Ju Gim, Chang-Hoi Ho, Jinwon Kim, Eun Ju Lee

**Affiliations:** 1 School of Earth and Environmental Sciences, Seoul National University, Seoul, Korea; 2 Climate Research Division, National Institute of Meteorological Sciences, Seogwipo-si, Korea; 3 School of Biological Sciences, Seoul National University, Seoul, Korea; Peking University, CHINA

## Abstract

Regional warming, owing to urbanization, leads to earlier spring phenological events and may expose plants to hard freeze damage. This study examined the influence of urbanization on the risk of frost damage to spring flowers in South Korea from 1973 to 2015. For the analysis period, we categorized 25 cities into two groups: those showing rapid population growth (rPG) ≥ 200,000, including 13 cities, and those showing no or decreased population growth (nPG), including 12 cities. We then investigated the time from the last frost dates (LFDs) in spring to the first flowering dates (FFDs) for each group. The rPG group experienced significant spring warming of 0.47°C per decade, resulting in earlier LFDs and FFDs. For this group, the advancement of LFD was more rapid than that of FFD, and the days between these two dates increased from 0.42 to 0.47 days per decade, implying a reduced risk of frost damage. Spring warming and the advancement of FFDs and LFDs were relatively small for the nPG group, and the LFDs were rather delayed. Consequently, the days between LFDs and FFDs were reduced from −1.05 to −1.67 days per decade, indicating an increased risk of frost damage. The contrasting changes in the frost-damage risk between the two city groups can be attributed to distinct urban warming at night, which makes the LFDs substantially earlier in the rPG group. Therefore, this study suggests that the warming associated with urbanization may lessen the risk of spring frost damage to plants in rapidly growing urban areas.

## Introduction

Climate change may substantially affect the composition and robustness of terrestrial ecosystems [1–3]. An important consequence of climate change is the altered risk of frost damage to plants [4–8]. Global warming causes an advance in spring plant phenological events [9–12] and the arrival of the frost-free period in spring [13–15]. Vulnerable plant tissues such as opening buds and expanding shoots develop in early spring [16, 17], which means that advancing phenological events can result in increased exposure of sensitive tissues to freezing temperature. Thus, the risk of frost damage increases or decreases depending on the degree of the advancement of spring phenological events and the arrival of the frost-free period [9, 18, 19]. If the phenological advancements occur more quickly than the frost-free period, the damage to plants could increase.

Urbanized areas have experienced localized climate change such that increases in surface air temperature are usually higher than those in the surrounding areas or rural regions [20–24]. Urbanization intensifies the urban canopy modification and the use of buildings and vehicles, which increases the land surface temperature in urban areas [25–27]. The magnitude of this urban warming is comparable to, or sometimes surpasses, the warming induced by increased greenhouse gas concentrations [24, 28–31]. Therefore, in urban regions, the timing of both plant phenological changes and frost events, and in turn the risk of frost damage to plants, can be altered by the warming induced by both urbanization and greenhouse gas emissions.

Urban warming shows a unique pattern in which more warming occurs at night than in the day [32–35], because heat added by human activity can easily accumulate on the land surface owing to the convectively stable atmosphere at night, which diminishes the vertical diffusion of the heat [20, 33, 36]. This distinctive urban warming could generate a unique pattern of changes in the frost-damage risk, although the effects of such urbanization remain unclear. Most studies on the changing risk of frost damage have assessed changes in climatic factors and the associated effects on the risks in specific regions [4, 6, 7, 18, 19, 37].

Previous studies on frost damage have reported a wide range of changes to the risks, according to regions. For example, Scheifinger et al. [37] reported that the time from a last frost event to the phenological spring phase has changed by −4 to +6 days per decade at 50 stations across Central Europe. This range may be partly attributable to varying magnitudes of urbanization because many weather observational stations are located in densely-populated districts for the convenience of use and maintenance of instruments. Therefore, for a more comprehensive understanding of the regional and continental changes in frost-damage risk, it is essential to quantify the influences of urbanization.

South Korea has experienced rapid economic growth in the last half-century due to the records set by a few economically more prosperous cities in which the magnitude of urbanization is greater than that of other cities [38]. The differences in warming among the cities have been recognized in previous research [31, 39, 40]. In this study, we compare cities with varying urbanization histories to identify the effects of urbanization on the risks of frost damage to spring flowers.

## Data and method

### First flowering and last frost dates in spring

**Fig 1:**
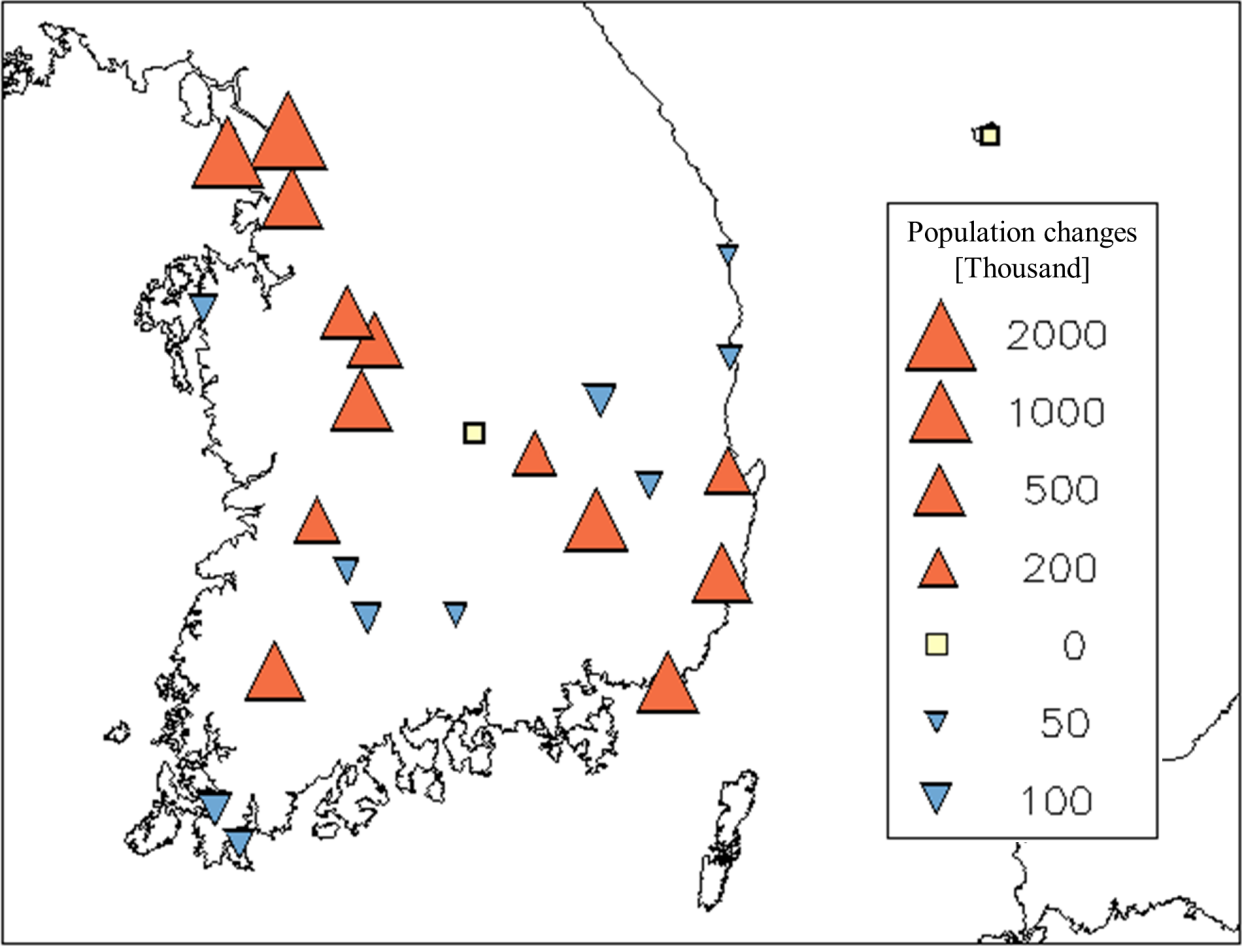
Locations and population changes in the 25 selected cities. The changes in population for 1975–2015 are denoted by direction and triangle size.

The Korea Meteorological Administration (KMA) has been recording meteorological and phenological variables in dozens of cities in South Korea. We chose cities that recorded daily temperature and the first flowering dates (FFD) of forsythia (*Forsythia koreana*) and azalea (*Rhododendron mucronulatum*) from 1973 to 2015, with a maximum of nine missing years. Cities with population increases of 0–200,000 were excluded in order to contrast clearly cities with rapid population growth (rPG, ≥ 200,000) from those with no or decreased population growth (nPG). Based on these criteria, 13 and 12 cities were classified as rPG and nPG, respectively. The significant effects of urbanization on the long-term warming trends have been reported for these 13 rPG cities in previous studies [31, 39]. In contrast, it is expected that the 12 cities in the nPG experience little or no urbanization, given a considerable reduction in the human population. In 10 of the 12 nPG cities, the population had reduced by more than half during the study period. Thus, a comparison between rPG and nPG groups could highlight the influence of the urban warming trend on the frost-damage risk. The selected cities for rPG and nPG are evenly distributed, geographically, in South Korea (Fig 1). Six cities in each group are located in coastal regions, and the remaining cities are located in inland regions. Although mountainous terrain is dominant in South Korea, all selected cities have an elevation below 250 m.

The gardens used for FFD observation are co-located with meteorological instruments at each synoptic station. The gardens are surrounded by grasses and short trees and are maintained to keep their original states under the management of the KMA. Determination of spring phenology of shrub species (i.e., forsythia and azalea) is based on monitoring of multiple plants of the target species in the station garden. The date on which more than 20% of buds of the plants fully open is set as the FFD for that species. Hereafter, we denote the FFD of forsythia and azalea as FFD_f_ and FFD_a_, respectively.

Last frost date (LFD) is defined as the last day in spring in which the daily minimum temperature drops below a critical temperature that can damage the flowers. Our analyses are based on two critical temperatures, −1°C and −2°C, considering that −2°C is reported as the critical temperature for flowers of shrub species [41, 42]. It is noted that the temperature of floral tissues can be lower than the surrounding air temperature owing to radiative cooling [43]. In addition to those temperature thresholds, we used adjacent temperatures in determining LFD, in order to reduce the dependency on the threshold selection. For example, we selected the last days of the daily minimum temperature below −0.5°C, −1°C, and −1.5°C and averaged those dates as the LFD with a critical temperature of −1°C. LFDs with thresholds of −1°C and −2°C are hereafter denoted as LFD_−1_ and LFD_−2_, respectively.

As an index of the risk of frost damage to flowers, the days from the frost date to the flowering date (DFF) were obtained by subtracting LFD from FFD, i.e., DFF is defined as the number of days between LFD and FFD. DFFs from four different combinations of LFD and FFD based on species and the threshold temperature were calculated for each city and are hereafter identified by two subscripts as DFF_f,−1_, DFF_f,−2_, DFF_a,−1_, and DFF_a,−2_, where the first subscript indicates the species (forsythia or azalea) and the second is the critical temperature of ‒1°C or ‒2°C.

### Models for flowering date

In order to identify the climatic factors that determine FFD, we employed three spring phenology models (i.e., spring warming, sequential, and alternating models) which are based on distinctly different assumptions of the plant’s response to climatic factors. These models assumed in common that a certain amount of thermal forcing (e.g., accumulated daily temperature above a threshold) is required for the occurrence of phenological events. However, in the spring warming model, the thermal forcing is the only climatic factor determining phenological timing, while chill days in addition to thermal forcing affect phenological timing in the sequential and alternating models. The sequential and alternating models differ in how they count chill days and how the chill days affect phenological timing. In these models, thermal forcing [44, 45] or combined thermal forcing and chilling days [46–48] are calculated according to the functional form of each model, and the date in which the state of forcing (*S*_*f*_) meets a critical accumulation of heat units (F*) is determined as the flowering date. We optimized the model parameters for each city with FFD records and found the best model in terms of the predictability of inter-annual variability and long-term trends.

In the spring warming model, thermal forcing is accumulated linearly with daily air temperature with a base temperature (*T_f_*):

$$S_{f}(t)=\sum_{t_{0}}^{t_{FFD}}\max\left(T_{air}-T_{f},0\right),$$ (1)

where *t*_0_ is the date in which thermal forcing begins to accumulate, *T_air_* is the daily air temperature, and *t*_FFD_ is the predicted flowering date in which *S_f_*(*t*) reaches a prescribed threshold of accumulated thermal units (F*).

In the sequential model, thermal forcing begins to accumulate after a critical sum of chill units (C*) are met. The state of chilling, *S_c_*(*t*), increases when the air temperature is colder than a base temperature for chill (*T_c_*):

$$S_{c}(t)=\sum_{t_{0}}^{t_{1}}\{\begin{array}{c} 1,T_{air}<T_{c}\\ 0,T_{air}>T_{c} \end{array},$$ (2)

where *t*_1_ indicates the date in which the required chilling days are met and thermal forcing begins to accumulate according to Eq (1).

In the alternating model, thermal forcing and chilling days accumulate from a single initiation date and with a single base temperature according to Eqs (1) and (2). The critical sum of heat units, F*, decreases exponentially as accumulated chilling increases:

$$S_{f}(t)\geq a+b∙exp(c∙S_{c}(t)),$$ (3)

where *a*, *b*, and *c* are parameters determining the shape of the exponential function, and *c* < 0.

## Results

### Distributions and changes in the first flowering and last frost dates

**Fig 2:**
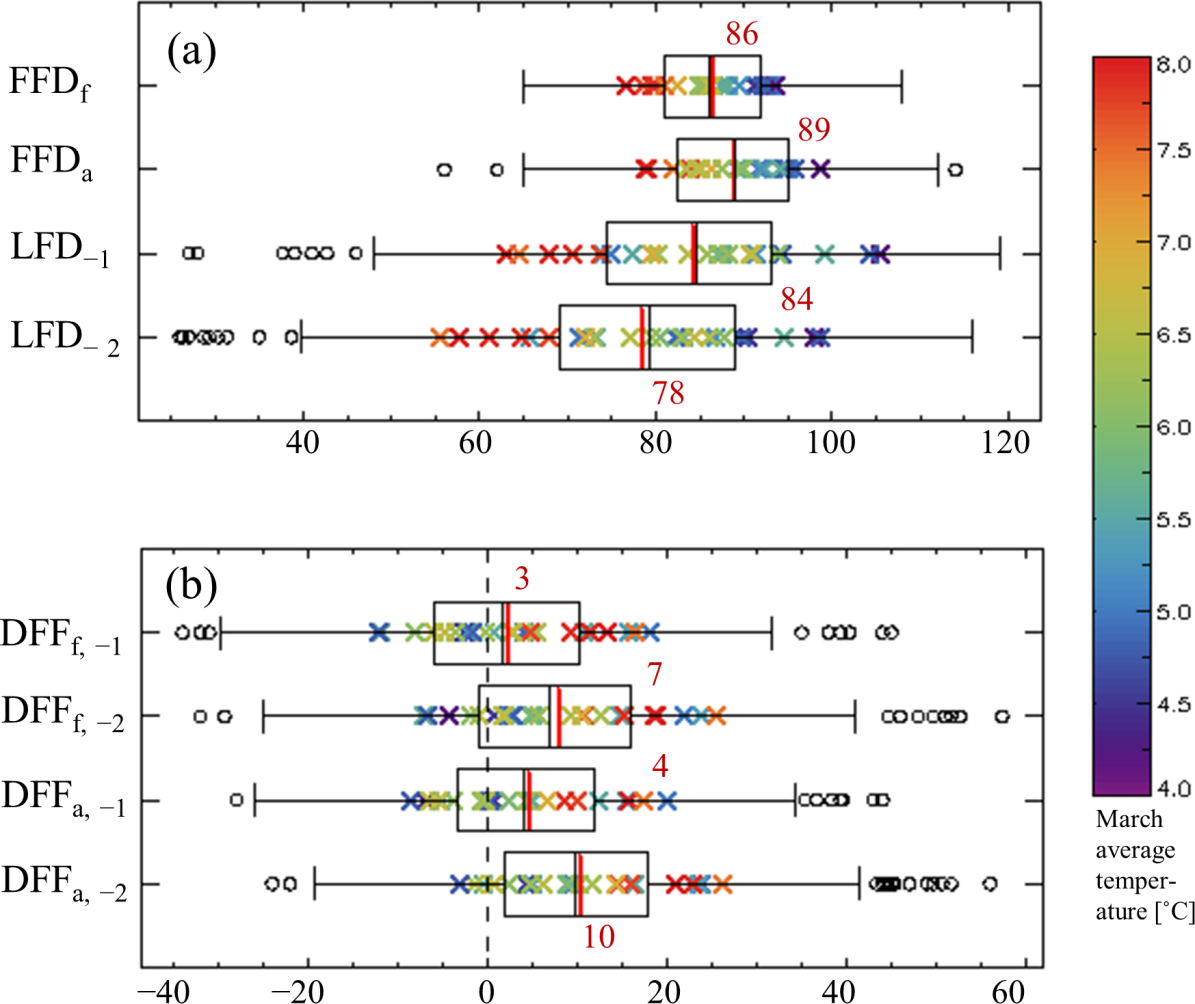
Distributions of the first flowering and last frost date and the days between them. The box-and-whisker plot of the entire records of each variable is depicted with total averages (red vertical bar) and city-averages (crosses) for 1973–2015. The color of the crosses represents the long-term average March temperature. Small circles are the outliers which are larger or smaller than median value by 1.5 times of interquartile range (i.e., 1.5 times of difference between 75^th^ and 25^th^ percentiles).

The FFDs of forsythia and azalea occur from early March to mid-April; on average, the 86^th^ and 89^th^ day of the year, respectively (Fig 2). More than half of the FFDs occur within two weeks from late March to early April. The long-term average of the FFDs ranges from the 75^th^ to the 100^th^ day of the year in the selected cities; there is a tendency for an early FFD in warmer cities. LFD_−1_ and LFD_−2_ occur on the 84^th^ and 78^th^ day of the year, respectively. Although LFDs precede FFDs by a week, on average, more than 25% of LFDs are later than the average FFD, owing to the wide variation in LFDs from mid-February to late April. This indicates that the possibility of frost damage is dependent on the city. The long-term average of LFD tends to be early in warmer cities, similar to that of FFD; however, the range of the average LFDs is wider than that of the FFDs, indicating higher sensitivity of LFD to temperature. In addition, no considerable differences were noted between the two city groups in the averages of FFDs or LFDs because there is no bias relating to regional climate or latitude of the rapidly growing cities (Fig 1).

Four different categories of DFFs—DFF_f, −1_, DFF_f, −2_, DFF_a, −1_, and DFF_a, −2_—indicated positive averages of 3 to 10 days (Fig 2). The DFFs for LFD_−2_—DFF_f, −2_ and DFF_a, −2_—showed the largest average values of 7 and 10 days, respectively, and those for LFD_−1_—DFF_f, −1_ and DFF_a, −1_—showed smaller averages of 3 and 4 days, respectively. Although positive averages indicate that the flowers are generally safe from frost damage, more than 25% of the DFFs were less than zero owing to the wide range in distribution from −30 to +40 days. This indicates that for one of the four cases, two spring flowers have been exposed to freezing conditions during the last decades. In addition, the DFFs tended to be smaller or even negative in colder cities.

**Fig 3:**
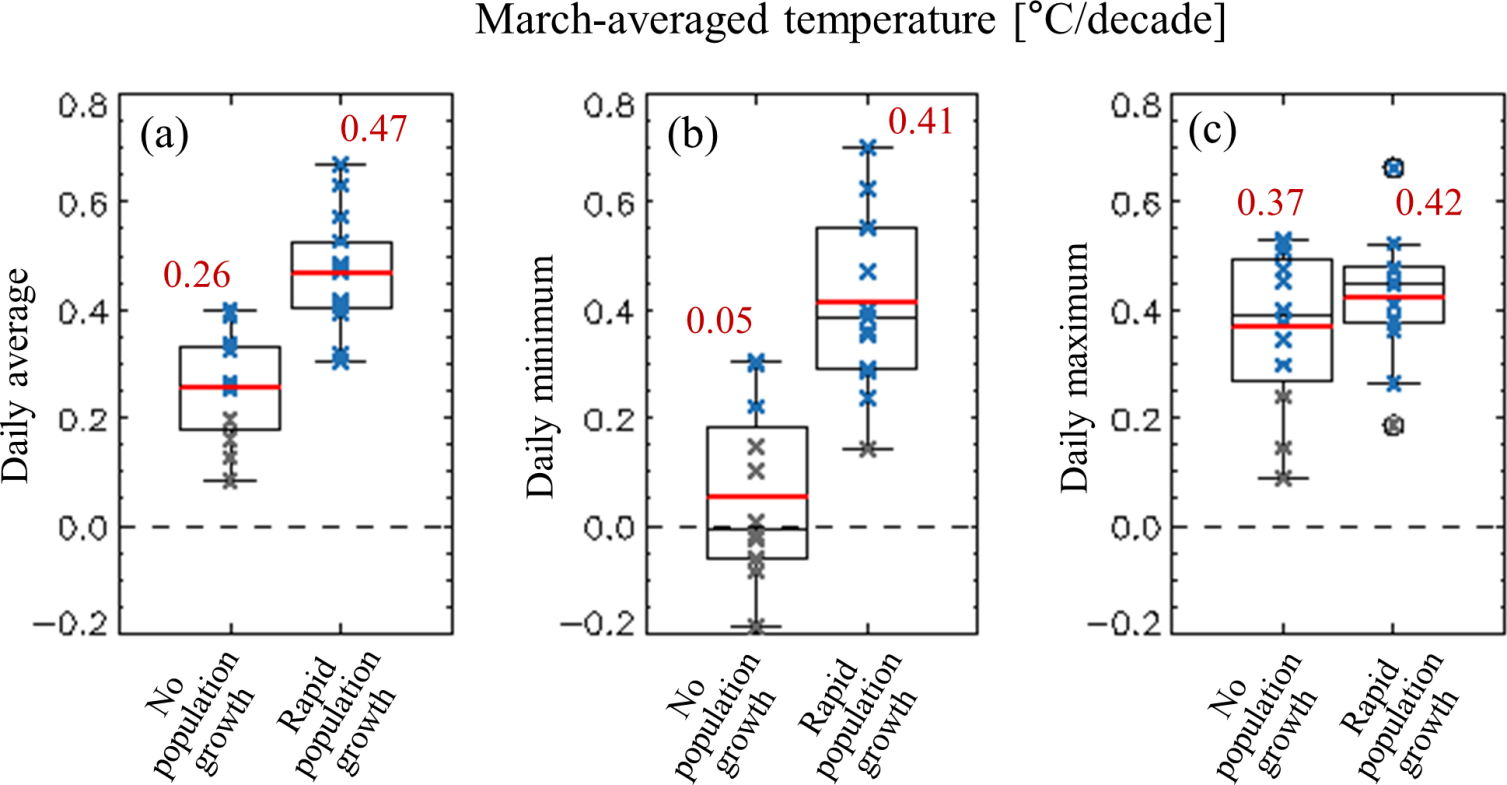
Long-term trends of March temperature in the 25 selected cities. The long-term trend of (a) average temperature in March, (b) average daily minimum temperature in March, and (c) average daily maximum temperature in March are shown. Statistically significant differences (*p* < 0.05)—significant enough to reject a null hypothesis of no change—are denoted by bold blue cross.

Most cities in South Korea showed significant warming trends. In particular, the warming in March in the rPG group, at 0.46°C per decade, appears to be greater than that in the nPG group, at 0.26°C per decade (Fig 3). This indicates that a considerable portion of the long-term warming trend of rPG cities is attributed to urbanization. In terms of the daily minimum temperature, the rPG cities showed much larger increases, at 0.41°C per decade, than the nPG cities, at 0.08°C per decade. The differences in daily maximum temperature increase were insignificant between these two city groups, at 0.42°C and 0.37°C per decade^−1^ in the rPG and nPG cities, respectively.

**Fig 4:**
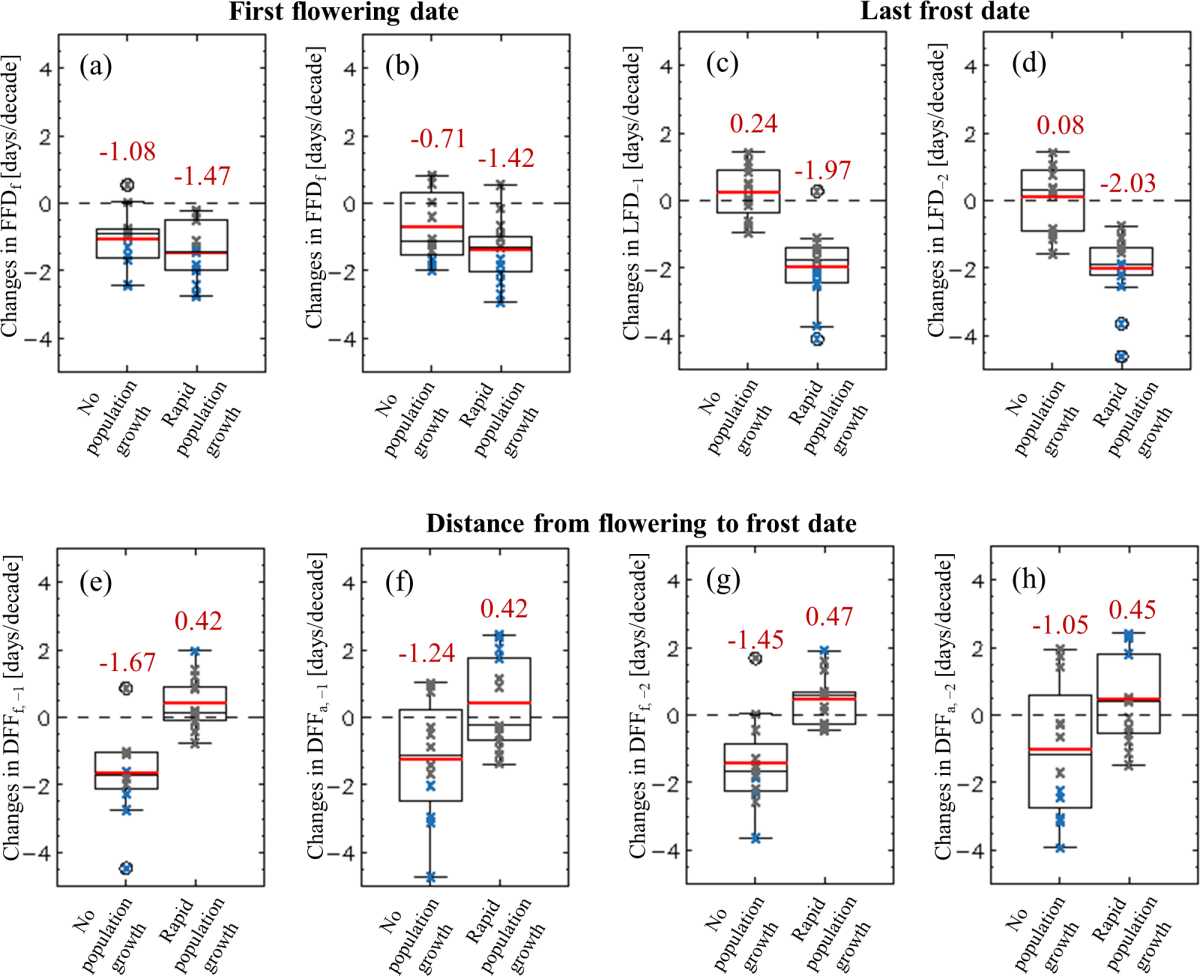
Long-term trends of first flowering date (FFD), last frost date (LFD) and the days between them. (a) FFD_f_, (b) FFD_a_, (c) LFD_−1_, (d) LFD_−2_. Also shown are the days between (e) FFD_f_ and LFD_−1_, (f) FFD_a_ and LFD_−1_, (g) FFD_f_ and LFD_−2_, and (h) FFD_a_ and LFD_−2_. The red number and red line indicate the average value of each city group. Statistically significant (*p* < 0.05) trends—large enough to reject a null hypothesis of no change—are denoted by bold blue crosses.

The FFDs of forsythia and azalea showed advancement in most cities and appeared to be greater for the rPG group, at −1.47 and −1.42 days per decade, respectively, than for the nPG group, at −0.71 and −1.08 days per decade, respectively (Fig 4). Statistically significant trends appeared in 5 and 3 of the 12 rPG cities for forsythia and azalea, respectively, and in 7 and 6 of the 13 nPG cities for forsythia and azalea, respectively. Moreover, long-term changes in LFDs showed clear separation between the two city groups. The LFD_−1_ and LFD_−2_ of the nPG cities were postponed by 0.24 and 0.08 days per decade, respectively, with no statistically significant trend shown. In contrast, those in the rPG group showed advancement by −1.97 and −2.03 days per decade, respectively, with statistical significance shown in both LFD_−1_ and LFD_−2_ for 6 of the 13 cities.

Consequently, the DFFs in nPG cities have been considerably shortened, which indicates an increased risk of frost damage to flowers (Fig 4). The DFFs for FFD_f_ shortened the most, at −1.67 and −1.45 days per decade for DFF_f, −1_ and DFF_f, −2_, respectively; those for FFD_a_ shortened less, at −1.24 and −1.05 days per decade for DFF_a, −1_ and DFF_a, −2_, respectively. In contrast, the rPG showed increases in DFF with averages of 0.42 to 0.47 days per decade for the four DFF categories, indicating reduced risk.

### Relationships of air temperature with FFD and LFD

We fitted the three spring phenology models with the observed FFDs to determine the optimal parameter set for minimizing the root mean square error (RMSE). The minimized RMSEs were considerably lower than the standard deviation of the recorded FFDs, at 5.88 and 6.59 days on average for forsythia and azalea, respectively (Table 1). In addition, the predicted FFDs explained about 60% of inter-annual variability on average, with a correlation coefficient (*r*) of about 0.8 and a model bias of less than one day. Among the three examined phenology models, the alternating model showed the lowest RMSE and the highest *r*. This result is consistent with a previous study showing that the alternating model performs best in explaining the variation in timing of budburst for *Rhododendron* spp. [49].

**Table 1:** Error statistics of the predicted first flowering date (FFD) of root mean square error (RMSE), correlation coefficient (*r*), and bias.

Phenology model	Species	RMSE	*r*	Bias
Spring warming	Forsythia	3.61 (0.72)	0.78 (0.08)	0.12 (0.42)
	Azalea	4.23 (0.97)	0.77 (0.07)	0.14 (0.36)
Sequential	Forsythia	3.65 (0.69)	0.78 (0.08)	0.00 (0.53)
	Azalea	4.17 (1.01)	0.78 (0.07)	0.06 (0.51)
Alternating	Forsythia	3.42 (0.58)	0.81 (0.06)	-0.02 (0.37)
	Azalea	3.96 (0.97)	0.80 (0.06)	0.05 (0.39)

The averages of the 25 target cities are listed, and the standard deviations are in brackets. The bias indicates the difference between the average of the reconstructed FFD and that of observations.

**Fig 5:**
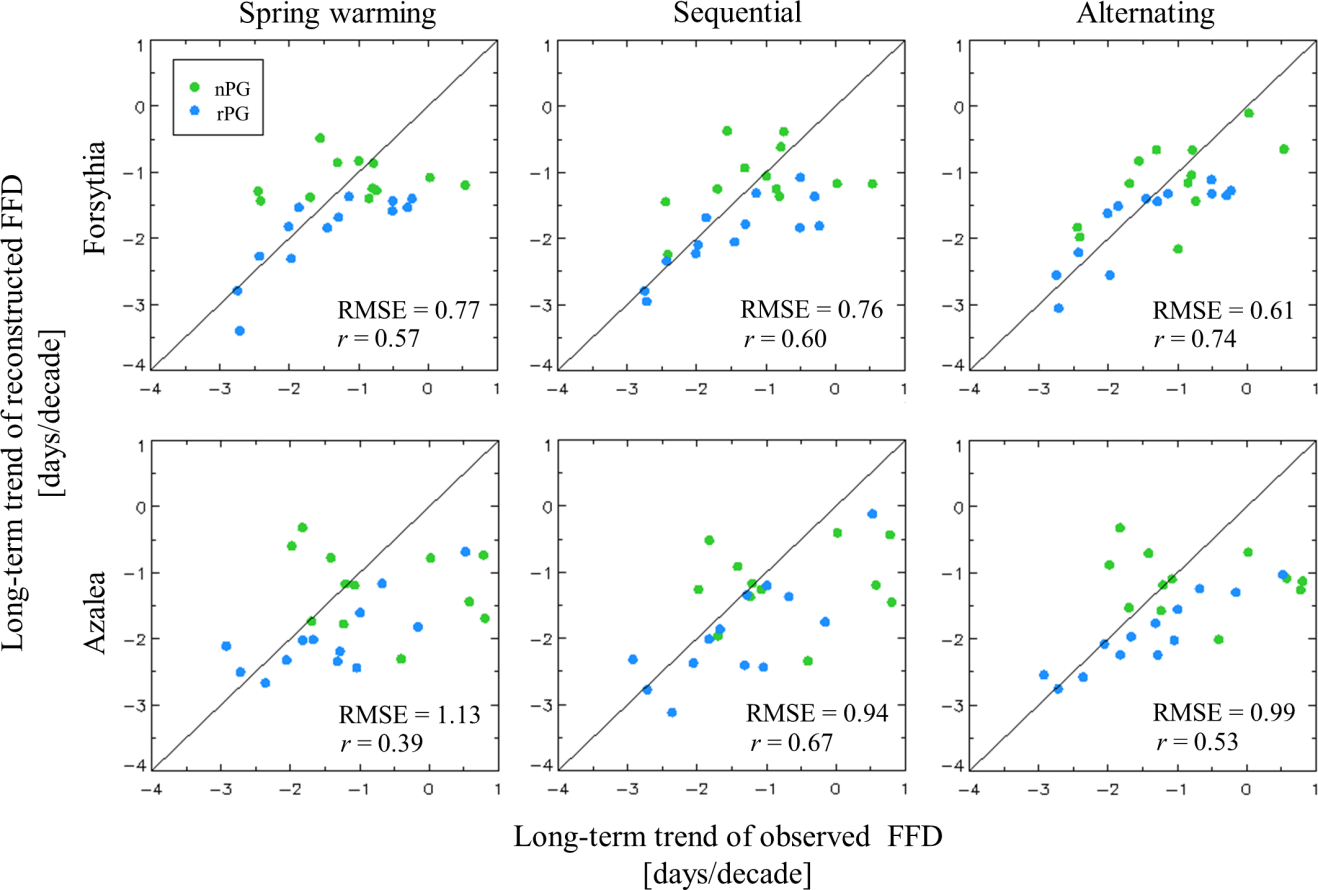
Long-term changes in FFD, comparing the observed and reconstructed values from the three phenology models in the 25 selected cities.

To assess the reliability of the long-term predictions, the magnitudes of the long-term changes were compared between the predicted and observed FFDs. For forsythia, these magnitudes were located close to the one-to-one line between predicted and observed FFDs; the RMSEs were less than 0.8 days per decade^−1^, and *r* was greater than 0.5 (Fig 5). The predictability for FFD_a_ was lower than that for FFD_f_, although the RMSEs of both species were lower than the standard deviation of the observed long-term changes which is 1.15 days per decade. Among these three phenological models, the alternating model outperformed other models, with smaller RMSEs of 0.15 days per decade for forsythia and azalea.

**Fig 6:**
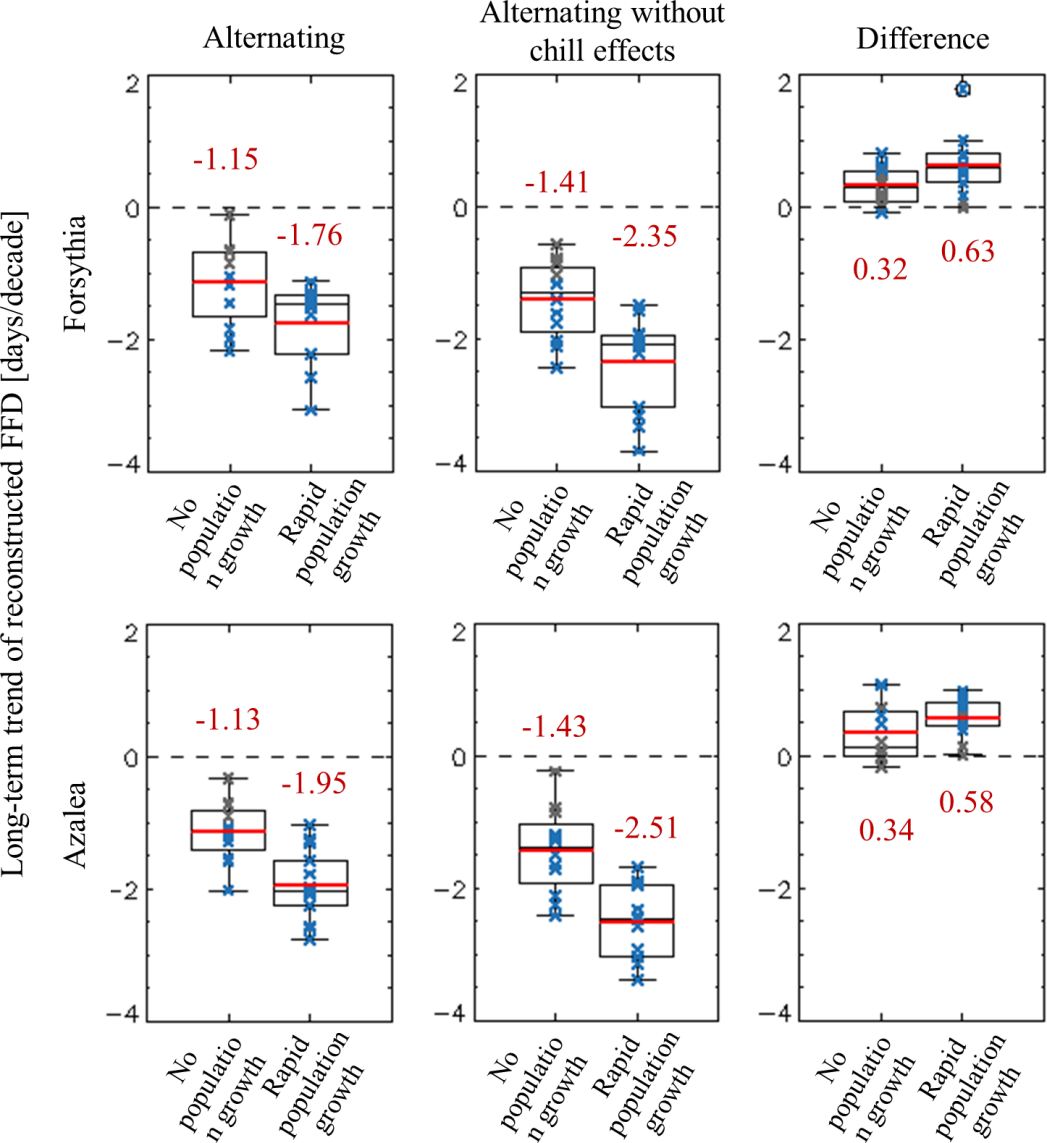
**Long-term changes in the predicted FFD of (a), (d) alternating and (b), (e) modified alternating models and (c), (f) the difference between them (alternating minus modified alternating).** Statistically significant (*p* < 0.05) trends)—large enough to reject a null hypothesis of no change—are denoted by bold blue crosses.

The alternating model uses the accumulated thermal and chill units to predict the spring phenology. We modified the alternating model to be insensitive to the changes in chill days in order to separate the contributions of chilling from those of thermal forcing to the long-term changes in FFDs. Then, we compared the results obtained with the original and modified alternating models. In the modified model, the required critical sums of thermal units are set to an average of the observed values. Without the dependency to chill in the modified model, the FFD advancements were larger than those of the original model (Fig 6). This indicates that the chilling effect weakened the FFD advancement under warming conditions. In addition, the differences in FFD advancement between the modified and original alternating models were larger in the rPG group, at 0.63 and 0.58 days per decade for FFD_f_ and FFD_a_, respectively, than those in the nPG group, at 0.32 and 0.34 days per decade for FFD_f_ and FFD_a_, respectively. This means that the weakening of FFD advancement owing to chill effects is larger in rPG cities than in nPG cites.

**Fig 7:**
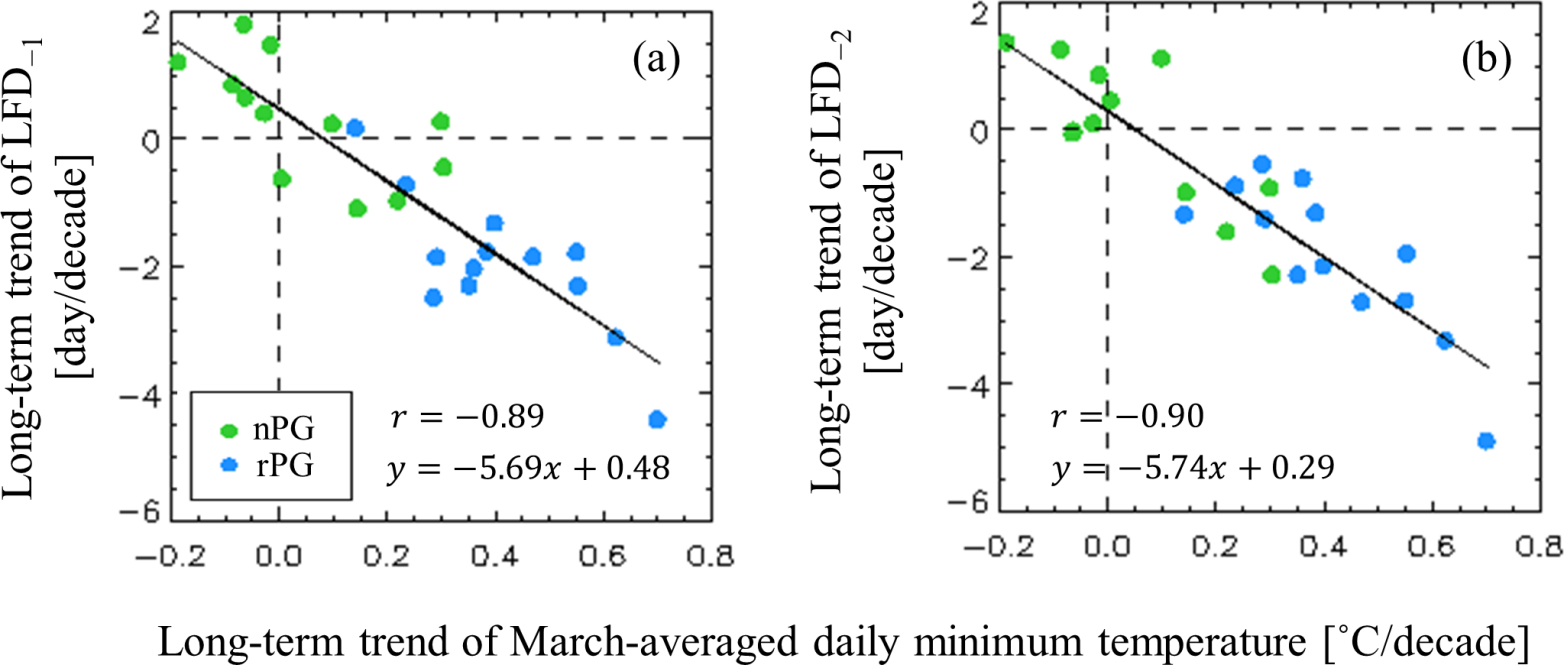
Long-term changes in (a) LFD_−1_ and (b) LFD_−2_ against long-term changes in the March-averaged daily minimum temperature.

The magnitude of long-term changes in LFD is predominantly determined by the trend of the daily minimum temperature in March. This conclusion is supported by three observations. Firstly, the advancement rates of LFDs and increase rates of the daily minimum temperature were closely correlated, at *r* = −0.89 and −0.90 for LFD_−1_ and LFD_−2_, respectively (Fig 7). Secondly, LFDs occurred earlier in several cities with increasing daily minimum temperatures, and LFD occurred later in the other cities with decreasing daily minimum temperatures. Thirdly, the slope of the changes in LFD against the warming, at −5.69 and −5.74 days °C^−1^ for LFD_−1_ and LFD_−2_, respectively, corresponded to the inverse slope of the seasonal increase in daily minimum temperature during the spring, at 5.92 days °C^−1^. Thus, we conclude that the distinct increase in the daily minimum temperature in the rPG group caused rapid advancement of LFDs and generated conditions that reduced the risk of frost damage.

## Discussion and conclusions

Climate change induced by human activity has intensified [50], resulting in adverse effects in the robustness and sustainability of ecosystems [1–3]. Urbanization, a predominant human activity at the regional scale, intensifies the urban heat island effects and increases urban land surface temperature [21] to cause significant long-term warming at night rather than during the day [33]. Although this particular warming pattern may exert a peculiar impact on ecosystems, which would be distinguished from the impact of global warming, the effects have not been systematically investigated. This study attempts to identify the urbanization effects on the risk of frost damage to spring flowers in South Korea.

In the rPG group, LFDs and FFDs became earlier due to considerable warming in spring. The advancement of the LFDs was relatively rapid compared to the FFDs, so DFFs increased with the rates of 0.42–0.47 days per decade. This indicates reduced risk from frost damage in the rPG group cities. In the nPG group, the LFDs showed no significant changing trend while the FFDs advanced significantly. This occurred because the daily minimum temperature, which is critical to changes in LFDs, increased marginally in the nPG cites even though the daily mean temperature increased significantly. Consequently, the DFFs for the nPG were shortened by −1.05 to −1.67 days per decade, indicating an increased risk of spring frost damage.

The discrepancy in the changes in risk between these two city groups is largely attributable to the distinct increase in the daily minimum temperature in the rPG cities (Fig 3), which caused substantial advancement in the LFDs (Fig 7), reducing the frost risk. The other main cause is the low sensitivity of FFDs to warming. As fewer chilling days accumulated owing to the warming, the sum of thermal units required for blooming increased, in turn lessening the susceptibility of FFDs to the warming (Fig 6). This weak susceptibility may be responsible for the insignificant differences in the advancement of FFDs between these two city groups (Fig 4) despite a warming trend 1.81 times stronger in rPG cities compared with the nPG cities.

In this study, we focused on two shrub species in which FFDs occur in early spring in order to examine the changes in frost-damage risk. The species with FFDs in late spring were not investigated because the flowers of those species are rarely damaged by frost. Although only two species were examined in this study, it is expected that the magnitude of the long-term change trend in the examined FFD may be a common trend for early spring phenology; at a minimum, the trends of the examined FFDs may not deviate considerably from the common trend. This expectation is based on Richardson and O’Keefe [49], indicating that changes in the phenological timing appeared to be similar for the phenological events occurring at a similar time (e.g., early spring).

For other species with relatively late flowering in spring, it is likely that frost-damage risk reduces more than, or increases less than, the changes in the risk for the species examined in this study. The species with relatively late spring phenological events (i.e., FFD or budburst) tend to adopt a less-risky life strategy and respond more conservatively to the environment [51]. Thus, the spring phenology of these species advances relatively weakly under warming conditions [52, 53]. For example, cherry and peach in South Korea—which are not included in this study owing to their late blooming and scarce occurrences of frost damage—advanced FFD under warming by a much smaller degree compared with the species showing early spring FFD [54]. Therefore, these late-flowering species may show a smaller difference in FFD advancement between the two city groups, which intensifies the risk-reduction effects of urbanization.

Based on previous studies, two critical temperatures, −1°C and −2°C, were used to determine the LFDs in this study. However, the critical temperature can be lowered in colder cities owing to higher cold tolerance of plants, caused by cold acclimation. The cold acclimation may have contributed to shorter or negative climatology of DFFs in the colder cities in this study (Fig 2). Thus, lower temperature thresholds might be more appropriate to identify the frost damage occurrences in colder cities. This study does not consider lower thresholds because it is difficult to measure the degree of acclimation and to determine the appropriate temperature thresholds. Furthermore, the change rates of LFDs with lower temperature were similar to those using original thresholds. The LFDs with two lower critical temperatures of −3°C and −4°C changed by 0.01 and −0.07 days per decade for the nPG group and by −2.04 and −2.27 days per decade for the rPG group, which is similar to the changes in LFD_−1_ and LFD_−2_ (Fig 4). Thus, consistent conclusions will be inferred in terms of the long-term changes in the risks regardless of the consideration of cold acclimation.

The degree of the influence of urbanization on frost-damage risk could vary among regional climates. One of the regional climatic factors affecting the urbanization influence is the degree of the seasonal increase in air temperature from winter to summer. In the regions in which air temperature increases relatively weakly from winter to summer, LFD advances more rapidly than that in other regions under the same degree of long-term warming [9]. For example, Scheifinger et al. [37] examined the changes in LFD in Central Europe and reported the advancement of LFD at a rate of about two days per decade. This rate is significantly higher than that determined for South Korea in this study (Fig 4) despite the relatively weak long-term warming in Central Europe. The high rate of FFD advancement in Central Europe can be attributed to a relatively warm winter, cool summer, and weak seasonal increasing trend of temperature from winter to summer. Thus, in the regions showing sensitive LFD advancement to warming, the advancement of LFD is distinctive in urbanized cities, which would cause a substantial reduction in frost-damage risk to spring flowers.
